# Transcultural adaptation and initial validation of Brazilian-Portuguese version of the Basel assessment of adherence to immunosuppressive medications scale (BAASIS) in kidney transplants

**DOI:** 10.1186/1471-2369-14-108

**Published:** 2013-05-21

**Authors:** Elisa de Oliveira Marsicano, Neimar da Silva Fernandes, Fernando Colugnati, Fabiane Rossi dos Santos Grincenkov, Natalia Maria da Silva Fernandes, Sabina De Geest, Helady Sanders-Pinheiro

**Affiliations:** 1 Renal Transplantation Unit, Division of Nephrology, Federal University of Juiz de Fora, Juiz de Fora, Minas Gerais, Brazil; 2 Núcleo Interdisciplinar de Estudos e Pesquisas em Nefrologia [NIEPEN], Juiz de Fora, Minas Gerais, Brazil; 3 Centre for Public Policy and Education Evaluation (CAED), Federal University of Juiz de Fora, Juiz de Fora, Minas Gerais, Brazil; 4 Institute of Nursing Science, University of Basel, Basel, Switzerland

**Keywords:** Patient adherence, Immunosuppression, Medication nonadherence, Transplantation, Validation

## Abstract

**Background:**

Transplant recipients are expected to adhere to a lifelong immunosuppressant therapeutic regimen. However, nonadherence to treatment is an underestimated problem for which no properly validated measurement tool is available for Portuguese-speaking patients. We aimed to initially validate the Basel Assessment of Adherence to Immunosuppressive Medications Scale (BAASIS^®^) to accurately estimate immunosuppressant nonadherence in Brazilian transplant patients.

**Methods:**

The BAASIS^®^ (English version) was transculturally adapted and its psychometric properties were assessed. The transcultural adaptation was performed using the Guillemin protocol. Psychometric testing included reliability (intraobserver and interobserver reproducibility, agreement, Kappa coefficient, and the Cronbach’s alpha) and validity (content, criterion, and construct validities).

**Results:**

The final version of the transculturally adapted BAASIS^®^ was pretested, and no difficulties in understanding its content were found. The intraobserver and interobserver reproducibility variances (0.007 and 0.003, respectively), the Cronbach’s alpha (0.7), Kappa coefficient (0.88) and the agreement (95.2%) suggest accuracy, preciseness and reliability. For construct validity, exploratory factorial analysis demonstrated unidimensionality of the first three questions (*r* = 0.76, *r* = 0.80, and *r* = 0.68). For criterion validity, the adapted BAASIS^®^ was correlated with another self-report instrument, the Measure of Adherence to Treatment, and showed good congruence (*r* = 0.65).

**Conclusions:**

The BAASIS^®^ has adequate psychometric properties and may be employed in advance to measure adherence to posttransplant immunosuppressant treatments. This instrument will be the first one validated to use in this specific transplant population and in the Portuguese language.

## Background

Transplant patients are expected to adhere to a lifelong therapeutic regimen designed to preserve long-term graft function and to reduce the risk of complications [1-4].

Adherence is defined by the World Health Organization as “the extent to which a person’s behavior – taking medication, following a diet, and/or executing lifestyle changes, corresponds with agreed recommendations from a health care provider” [5]. In the setting of transplantation, a recent consensus conference stated nonadherence (NA) as “deviation from the prescribed medication regimen sufficient to adversely influence the regimen’s intended effect” [6].

Kidney transplant (KTx) is the most widely performed transplantation procedure worldwide [7]. In 2010, 16,898 KTx were performed in the United States [7]. In the same period, 4,630 KTx were also done in Brazil, the second country in absolute numbers of KTx in the world [8,9]. Some reports have indicated KTx recipients to be the most nonadherent among transplant patients [3,10]. Indeed, a recent meta-analysis study revealed that the magnitude of NA to immunosuppressives in KTx recipients was as high as 35.6 cases per 100 patients per year, indicating a prevalence expressive superior than the overall population of solid organ transplant recipients, which was 22.6 cases per 100 patients per year [3]. An estimated 15% to 60% of late acute rejections and 5% to 36% of graft losses were associated with NA in renal transplant patients [11]. These data are disturbing given that the odds of graft failure increases by approximately seven-fold in nonadherent renal transplant recipients compared with adherent subjects [11].

Given the high number of organ transplantations performed in Brazil, especially kidney transplants, it is clear this community needs effective tools to identify patients at risk of NA. Detection of NA is the first step in identifying patients at risk who then can be targeted through preventive and restorative interventions in the transplant population [4,12]. Several methods of NA detection in transplant patients have been suggested in the literature, such as blood assay, pill count, electronic monitoring, and prescription refill. These methods can best be used in combination to maximize the sensitivity and accuracy of adherence measurement, this methodology is called ‘triangulation’ [5,13]. Despite the known limitations, regarding the use of self-report instruments to measure immunosuppressives NA, including underreporting and social desirability bias, self-report instruments are cheap, easy to use, uncomplicated to score, and applicable as part of a combined diagnosis strategy. Several self-report methods have been proposed to measure posttransplant immunosuppressives NA elsewhere [14]. However, to date, no self-report instrument to assess NA to immunosuppressive therapy has been validated for use in Brazilian Portuguese-speaking patients.

Thus, we searched for a self-report method that we could validate for this purpose and opted for the Basel Assessment of Adherence to Immunosuppressive Medications Scale (BAASIS^®^) prompted by a review published by Dobbels et al. This review favored the BAASIS^®^ as one of the most optimal self-report instruments for measuring NA in transplantation [14]. The BAASIS^®^ assesses relevant dimensions of immunosuppressive drug use, i.e. taking adherence, timing adherence, drug holidays and dose reduction in a recording fixed time of the last four weeks. It is also comparatively shorter than other self-report methods suggested for assessing NA in transplant patients [14]. More importantly, the instrument has already been used in research and clinical practice and has been chosen by the Transplant360 Task Force to disseminate, albeit without being fully validated [14-16]. Its validation for transplant populations is in progress in several projects by the Leuven-Basel Adherence Research Group (personal communication: Sabina De Geest, University of Basel, Dec 2012). Moreover, the predictive validity of the BAASIS^®^ in adult liver transplant recipients had been recently established (Paolo De Simone, University of Pisa, manuscript in preparation). The validation process lacks of standardization and some controversies about the minimal content of validation studies still exist. Besides, a primary test of psychometric properties allows the clinical application and further predictive analyses [17-19].

Therefore, the aim of this study was to transculturally adapt and initially validate the BAASIS^®^ to measure immunosuppressant NA in Brazilian Portuguese-speaking transplant patients.

## Methods

### Study design

**Figure 1:**
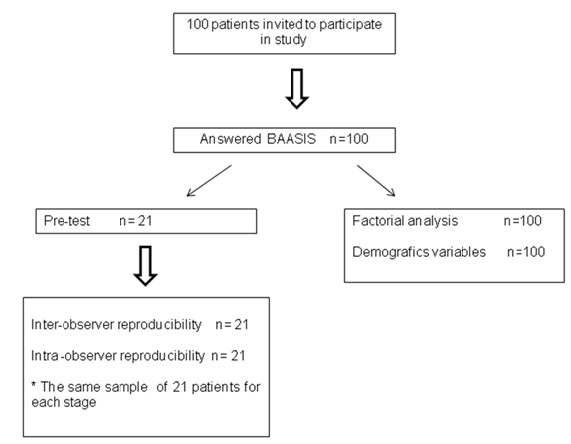
Design of the study.

To initially validate the English version of the BAASIS^®^’ in Brazil, we conducted a single-center cross-sectional study. Patients were recruited between May 1, 2010 and December 1, 2010 (Figure 1).

### Sample, setting and data collection

We evaluated a convenience sample of 100 KTx patients who were recruited from the outpatient facility of the Núcleo Interdisciplinar de Estudos, Pesquisas e Tratamento em Nefrologia of the Federal University of Juiz de Fora. In the transcultural adaptation and in reproducibility tests, we evaluated a subgroup of 21 subjects out of the 100 included in this study in these patients, measurements were repeated after seven days [17,18,20] (Figure 1).

Patients were included in the study based on the following criteria: being at least 18 years old, more than 1 year after transplantation, and willing to participate in the study by signing an informed consent form approved by the local Ethics in Research Committee of University Hospital of Federal University of Juiz de Fora (approval number - 0068/2010). The exclusion criteria were as follows: retransplant, dependence on others for medication management and illiteracy [10,15].

The instruments BAASIS^®^ and Measure of Adherence to Treatment (MAT) were administered to included patients during their regular consultation visit, by transplant trained nurse interviewers.

### Variables and measurements

Demographic and clinical variables were retrieved from the medical files.

The authors of the BAASIS^®^ provided permission to translate the copyrighted instrument into Portuguese. It consists of a four-item questionnaire measuring NA to immunosuppressives over the past four weeks. It assesses the crucial dimensions of drug-taking NA (omission of single doses), drug holidays (omission of successive doses), timing NA (timing deviations > 2 h) and dose reductions. Responses are given on a six-point scale: never (0), once per month (1), every second week (2), every week (3), more than once per week (4), and every day (5). Patients with any deviation, namely an answer different from “never” in any of the four questions, are considered as nonadherent (Table 1). The interview in the English version of BAASIS ^©^ starts with a table that assesses the transplant patient’s medication regimen. The table is filled out by the health-care professional and the patient together. The name, dose and dosing frequency and times of each drug are noted [14,15,21].

**Table 1:** **The two versions of the BAASIS**^**© **^**scale, original in English and final proposed version**

**Original version**	**Portuguese version**
The Basel Assessment of Adherence with Imunossupressive Medication Scale (BAASIS)	Escala Basel *Para*^a^ Avaliação De Aderência a Medicamentos Imunossupressores (BAASIS)
1) Do you recall not having taken your immunosuppressive medications (give name of drugs) some times in the past 4 weeks?	1) Você se lembra de não ter tomado seus remédios imunossupressores (dê o nome dos remédios) alguma vez nas últimas 4 semanas?
2) Have you skipped several consecutive doses of your immunosuppressive medications in the past 4 weeks?	2) Você deixou de tomar *várias*^a ^doses consecutivas de sua medicação imunossupressora nas 4 últimas semanas?
3) Do you recall having taken your immunosuppressive medications with more than 2 hours time difference from the prescribed dosing time, in the past 4 weeks?	3) Você se lembra de ter tomado seus remédios imunossupressores com mais de 2 horas de diferença em relação ao horário prescrito, nas últimas 4 semanas?
4) Have you reduced the prescribed amount of your immunosuppressive medications during the past 4 weeks?	4) Você tomou uma dose menor do que a dose prescrita *pelo seu médico*^a^, nas últimas 4 semanas?
( ) yes ( ) no	( ) sim ( ) não
Could you tell me how often this happened: ^c^	Você pode me dizer com que freqüência isto aconteceu: ^c^
( ) Never	( ) Nunca
( ) Once a month	( ) Uma vez no mês
( ) Every two weeks	( ) A cada duas semanas
( ) Every week	( ) Toda semana
( ) More than once a week	( ) Mais de uma vez por semana
( ) Every day	( ) Todo dia

^a ^Modifications from the transcultural adaptation are highlighted in italic and underlined.

^b ^© University of Basel, Leuven-Basel Adherence Research Group, Institute of Nursing Science, University of Basel, Belgium, 2005. Permission & conditions to use the BAASIS^® ^can be obtained from sabina.degeest@unibas.ch.

^c ^The Likert scale should be applied to each of the four items if the answer is yes.

### Validation procedures

For the BAASIS^®^ initial validation, we followed the recommended international methods for transcultural adaptation [17,18] and subsequently corroborated the psychometric properties of the resultant transculturally translated instrument for adherence to immunosuppressive medications in KTx patients [22,23].

#### Transcultural adaptation

##### Translations

The BAASIS^®^ was first individually translated to Portuguese by two Portuguese-speaking professionals. The two translations were then compared and synthesized, producing the first version of the instrument. Next, the first version was back-translated into English by two other fluent speaking translators. The researchers synthesized the new English version and compared the result with the original so that any inconsistencies could be corrected. The corrected version was finally approved by the translators, producing the second Portuguese version of the questionnaire [17,18].

##### Expert committee

A five-member committee of bilingual health professionals revised versions 1 (in Portuguese) and 2 (in English) taking into account the semantic, idiomatic, and conceptual equivalence to identify possible confounders in the instrument. We then evaluated the version generated by the expert committee and implemented their suggestions to create the third version.

##### Pretesting

The third version was applied to the subgroup of 21 subjects who, after application, were asked about their understanding of the instrument content (Figure 1). Based on their responses, the third version was revised, producing the fourth and final version of the instrument in Portuguese (Table 1).

#### Psychometric properties

##### Reliability

Internal consistency was tested by calculating the Cronbach’s alpha for all the 100 study participants [23,24]. In those who were submitted to two tests, as part of test-retest reability, we considered the first result (Figure 1). Test-retest reliability was assessed using repeated assessments of the instrument in the subsample of 21 patients. Assessments were performed by the same observer to test intraobserver reproducibility and by different observers to test interobserver reproducibility/interrater reliability over a seven-day period. We then calculated the variance between the measurements (interobserver and intraobserver) by applying the Gage’s variance partition method. Kappa concordance coefficient was used to evaluate the intraclass correlation of the NA diagnosis. We also calculated the test-retest categorical agreement [25].

##### Validity

Content validity was determined during the transcultural validation of the instrument, using questions presented to different referees or experts who, in turn, identified the relevant goals to be measured and analyzed as representative for each item. The inconsistencies identified by the committee were all accepted by the investigators, generating the third version of the instrument [14,22].

Criterion validity was verified by correlating the transculturally adapted BAASIS^®^ with the MAT scale, using the Spearman’s correlation coefficient. We chose the MAT, a medication medication self-report scale for broad use in medical populations, because it is the only NA instrument adapted and validated in Portuguese which approximates our objectives, yet admittedly not being specifically developed for transplant patients. It consists of a seven items referring to taking, timing, and dose reduction, without a fixed recording period. Any answer different from “never” or “rarely” classifies a patient as nonadherent [26].

Construct validity, which refers to the ability of each question to measure a specific aspect of a more general construct, was verified through Exploratory Factorial Analysis, using principal components estimates for factor loadings and the Kaiser-Guttman criterion for dimensionality assessment (eigenvalue ≥ 1) [27].

### Statistical procedures

Baseline characteristics were described as mean ± standard deviation and categorical variables were represented as frequencies.

As detailed in each specific section above, intraobserver and interobserver variances by Gage’s variance partition method, Kappa concordance coefficient, test-retest categorical agreement, Cronbach’s alpha, Spearman’s correlation, Exploratory Factorial Analysis through principal components analysis and the Kaiser-Guttman criterion were performed using the Statistical Package for the Social Sciences 15.0 (SPSS Inc., Chicago, IL, USA).

## Results

### Sample characteristics

Sixty-five percent of the patients were male, 72% were white, and the mean age was 45 ± 13.5 years. Regarding the educational level, 45% completed primary school; 25%, secondary school and 30%, higher education. Most patients (89%) received their graft from a living donor. Mean posttransplant time was 72.3 ± 44.4 months. Average creatinine level was 1.56 ± 0.56 mg/dL. Only 30% lived in the same city of the transplant center (Table 2). Applying the final version of the transculturally adapted BAASIS^®^, 34% of the patients were considered nonadherent.

**Table 2:** Demographic characteristics

**Characteristics**	**%/N**
**Male gender**	65% (65/100)
**White race**	72% (72/100)
**Age** (years)	45 ±13.5
**Education level**	
Primary school	45% (45/100)
Secondary school	25% (25/100)
Higher education	30% (30/100)
**Mean post-transplant time **(months)	72.3 ± 44.4
**City of origin**	
Transplant center	30% (30/100)
Other cities in the same state	43% (43/100)
Other state	27% (26/100)

### Transcultural adaptation

The process of transcultural adaptation was the first step for validation of the BAASIS^®^ for the purpose of measuring the posttransplant patients’ adherence to immunosuppressive treatments. The process involved translation, synthesis, back translation, expert committee evaluation, and pretesting, as described in the Methods section [17,18]. The translation, back translation, and synthesis were uneventful. The expert committee proposed only three simple modifications concerning the instrument: the switch in the title (*of* to *for*, or *“para”* in Portuguese) thus clarifying the purpose of the scale; reintroduction of the word *several* (in Portuguese: *“várias”*) in question 2, which was omitted during the translating process; and the introduction of the Portuguese expression *“pelo seu médico”* (in English: *“by your doctor”)* in question 4 to assure that skipping was arbitrary (Table 1).

None of the 21 participants of the pretest had doubts of the meaning of or constraints in understanding the content when answering the third version of the instrument. The Portuguese version of the instrument was thus originated (Table 1).

### Psychometric properties

To complete the initial validation of the transculturally adapted BAASIS^®^ instrument, we next assessed its reliability and the three types of validity (content, criterion, and construct).

#### Reliability

The reliability of the instrument, assessed through intraobserver and interobserver reproducibility, indicated a very low measurement error of 0.101, which is equivalent to a measurement variance of 0.010. The interobserver and intraobserver errors obtained are presented in Table 3. Cronbach’s *alpha* was 0.70, indicating moderate internal consistency. For the Test-Retest subset, Kappa coefficient was 0.88, the agreement was 95.2%, both indicating almost perfect agreement.

**Table 3:** Reliability of the transculturally adapted BAASIS^®^, tested by intra and inter-observer reproducibility measurements

**Measurement**	**Variance**	**Standard deviation**
Total error	0.010	0.101
Intra-observer	0.007	0.084
Inter-observer	0.003	0.055

^a ^For the inter-observer reproducibility was calculated applying the adapted version in a sample of 21 subjects by the same observer at baseline and then by another one 7 days after. For the intra-observer we used the same procedure without changing the observer at day seven. Differences between scores obtained are scored as variance.

#### Validity

##### Content validity

Content validity was undertaken during the transcultural adaptation stage, using the proposals of the expert committee, and described above when we presented the results regarding the transcultural adaptation stage. The referees suggested modifications only for questions 2 and 4. There were no suggestions for other questions. All the inconsistencies were minor and promptly incorporated into the third version, which was finally approved by the committee (Table 1).

##### Criterion validity

Criterion validity was determined through correlations with existing instruments. A Spearman’s coefficient of 0.65 (p < 0.001) was obtained when the transculturally adapted BAASIS^®^ was correlated with the MAT.

##### Construct validity

Applying principal components analysis and Kaiser-Guttman criterion, we found a scree plot in which only one factor had an eigenvalue of 1.8 and accounted for 60% of the total variance, thus, we assumed only this factor in the subsequent approaches. The exploratory factorial analysis demonstrated that questions 1, 2, and 3 of the transculturally adapted instrument had adequate factorial loads (correlations between the answers to the questions and the general score of the instrument), that is, close to 1, similar to one another and higher than 0.4. In contrast, question 4 did not have a good factorial load. When question 4 was excluded, any important modification in the factorial loads of the other questions was observed. So, the question was retained in the BAASIS^®^ Portuguese version as in the original English version (Table 4).

**Table 4:** Factorial analysis of the transculturally adapted BAASIS^®^

**Questions**	**Factorial loads**	
	**All questions**	**Without question 4**
One	0.76	0.76
Two	0.81	0.83
Three	0.69	0.71
Four	0.35	-

^a ^The table presents the individual factorial loads of each question of the transculturally adaptation of BAASIS^® ^for measuring patients’ adherence to immunosuppressant treatments.

## Discussion

The aim of this study was to translate the BAASIS^®^ in a culturally sensitive way and to evaluate its psychometric properties in view of content-, construct- and criterion-related validities in adult renal transplant recipients. The BAASIS^®^ was chosen as the most promising self-report method of assessing patient adherence to immunosuppressive treatment after transplantation in Brazil. While there are many self-report instruments to assess adherence to immunosuppression, they have not been validated for use in Brazil, which is second in the world in terms of absolute numbers of KTx [9].

Because self-reporting tends to underestimate NA, selection of the ideal instrument is a crucial step for the identification of nonadherent patients [14,22]. The Transplant360 Task Force identified three self-report NA measurement tools that could be adapted to transplant clinical practice: the BAASIS^®^, the Medication Adherence Self-Report Inventory, and the Brief Antiretroviral Adherence Index Questionnaire [14]. Indeed, all these instruments evaluate both the drug taking and the regularity of medication intake and are considered easy to use and score [14]. Nevertheless, we chose to validate the BAASIS^®^ because it is concise, it seemed to comprise the relevant questions, and from the three proposed instruments, it is the only one already in use for transplant patients in other countries [15]. Moreover, we recently learned that the predictive validity of the BAASIS^®^ had been established in an adult liver transplant population (Dr. Paolo De Simone, University of Pisa, manuscript in preparation), further underscoring the value of our choice. We transculturally adapted and validated the BAASIS^®^ for the assessment of NA to immunosuppressant treatment in Brazilian KTx patients, using a previously described protocol [17,18].

Brazil is a continental country. In 2010, its population reached 190,732,694 despite having many regional disparities. The Brazilian health system is government managed and serves majority of the population [9]. Our study sample reflects many demographic patterns of the Brazilian KTx population, since it consisted of young Caucasian individuals, between 40–45 years, most received living grafts [8,28,29]

The BAASIS^®^ transcultural adaptation was fully accomplished by employing an expert committee [17,18]. The five participating professionals analyzed the original instrument, the translation, the back translation, and their proposals contributed to the development of the final version of the instrument. These steps have been successfully employed for other transcultural adaptations of other scales [30-32]. As the expert review resulted in a few changes, we could speculate that the scale is objective and that the questionnaire could be easily applied to the transplant population.

The result of the psychometric properties analysis demonstrated that the transculturally adapted BAASIS^®^ was a reliable instrument for measuring NA to immunosuppressant treatment in the Brazilian KTx patients investigated in this study. In fact, the intraobserver and interobserver reproducibility tests revealed little variability. Moreover the test-retest agreement and intraclass Kappa correlation were excellent. The results obtained were similar and therefore reproducible regardless of who applied the questionnaire or when it was applied, as indicated by the data. In addition, the Cronbach’s alpha was 0.70, indicating satisfactory internal consistency and good measurement performance [22,24]. This level of internal consistency was also reported for other self-report instruments for measuring NA to immunosuppressants or other medications [33,34].

As for content validity, potential misunderstanding problems were mitigated during the expert committee evaluation stage and changes were integrated in the transcultural adaptation step, as previously discussed.

For criterion validity analysis, the instrument to be validated must be compared with a gold standard [17,18,22] yet a gold standard for medication adherence measurement is still lacking [13,35]. Some studies have suggested that electronic monitoring methods should be adopted as the standard method as it has superior sensitivity and allows visualization of medication-taking dynamics [22,36,37]. Unfortunately, due to its considerable cost we were unable to use this method in our study. Moreover, electronic monitoring remains an indirect method as ingestion is not proven. Using immunosuppressives blood levels are another option, even taking into account its limitations based on individual pharmacokinetic variations. In our study population, we found a very low frequency of unacceptable immunosuppressive target levels, 7% (data not shown), we then considered this method to be inadequate to use for validation. Other methods such as pill count or use of clinical outcomes are unreliable to use as a comparison standard. In Brazil, we do not have a centrally managed database with prescription refills, limiting the use of this option for validation purposes.

Thus, we correlated the transculturally adapted BAASIS^®^ with the MAT. Despite the MAT being a general adherence assessment scale and the BAASIS^®^ being an immunosuppressive-specific instrument, we obtained a Spearman correlation of 0.65, indicating acceptable concurrent validity. It must be taken into account that if the correlation was too high, the transculturally adapted BAASIS^®^ validation would be immaterial because it would mean that a validated instrument to measure NA to immunosuppressant drugs was already established. Therefore, it seems that the transculturally adapted BAASIS^®^ presented herein is more appropriate to measure NA to immunosuppressant treatment than MAT.

We next performed Factorial Exploratory analysis to assess the construct validity of the transculturally adapted BAASIS^®^. Because high factorial loads were obtained in only one factor, we assumed to be the construct - adherence, the unidimensionality of the instrument was demonstrated [25]. Despite question four having the lowest load, pointing to its less relevant discriminatory power, its exclusion in a further exploratory analysis obtained the same unidimensional positive pattern. We suspected that the universal access to immunosuppressive medication provided by the public health system in Brazil could be a possible determinant of these results. We decided to retain question 4, as in the original version, thus avoiding the creation of a totally new instrument. However, the transculturally adapted BAASIS^®^ version should be further applied in a larger Brazilian patient population to verify if this pattern of question four will repeat.

One limitation of our study is that we only examined the transculturally adapted BAASIS^®^ in the context of assessing NA to immunosuppressant treatment in KTx patients mostly receiving living grafts. It is reasonable to think that the instrument initially validated herein will be also useful to assess NA of patients undergoing other types of transplants. However, future studies will be necessary to confirm this supposition. Moreover, the nonadherence is a complex phenomenon as well its diagnosis. We have been looking for a gold standard method for this purpose. Here, we propose to transculturally adapt and test the psychometric properties, i.e., validation procedure, of the BAASIS^®^. In criterion validation we are supposed to use the gold standard test or as in our case, a similar instrument. As discussed above we opted to use the MAT, which have has some limitations but it is suitable for the process, based on international guidelines for instrument development and testing [17,18,22]. “However, controversies about the content and methodological quality of the test validation process remain. Terwee and colleagues suggested a consistent proposal to improve the quality of health status questionnaires and to design validation studies. They enrolled eight psychometric properties and explicited ranking criteria for each property, albeit most of the recommendations are directed to questionnaires with many items. We are glad to report that applying the proposed guidelines, our validation process achieved very reasonable scores, achieving a positive ranking in four and an intermediate ranking in one, of five applicable items. However, some of the not graded psychometric properties, as responsiveness and interpretability, require further longitudinal studies to complement the initial validation we performed in the present study [19,38].

The consequences of NA to immunosuppressives are substantial, not only clinically but also economically, jeopardizing transplant outcomes [4,5,39,40]. Thus, there is a critical need to identify, through specific validated instruments, as those presented in this study, to provide interventional strategies for nonadherent transplant recipients and further to prevent undesirable adverse events.

## Conclusions

The transcultural adaptation of the BAASIS^®^ presented herein was performed according to international standards. This transculturally adapted and initially validated instrument is the first simple and easy-to-apply self-report method of detecting immunosuppressant NA in transplant patients in Brazil and other Portuguese-speaking countries.

## Abbreviations

BAASIS: The basel assessment of adherence to immunosuppressive medications scale; MAT: Measure of adherence to treatment; NA: Nonadherence; KTx: Kidney transplant.

## Competing interests

The authors declare that no competing interests exist.

## Authors’ contributions

*EOM:* Participated in the study design, review, analysis of the findings, writing of the paper, and approval of the manuscript. *NSF:* Participated in the study design, review, analysis of the findings, writing of the paper, and approval of the manuscript. *FC and NMF*: Participated in the study design, analysis of the findings, review of the paper, and approval of the manuscript. *FRSG and SG:* Participated in the study design, review of the paper, and approval of the manuscript. *HSP:* Participated in the study design, review, analysis of the findings, writing and reviewing of the paper, and approval of the manuscript. All authors read and approved the final manuscript.

## Pre-publication history

The pre-publication history for this paper can be accessed here:

http://www.biomedcentral.com/1471-2369/14/108/prepub

## References

[B1] Meier-Kriesche HU Schold JD Srinivas TR Kaplan B Lack of improvement in renal allograft survival despite a marked decrease in acute rejection rates over the most recent era Am J Transplant 2004 4 3 378 383 10.1111/j.1600-6143.2004.00332.x 14961990

[B2] Denhaerynck K Dobbels F Cleemput I Desmyttere A Schäfer-Keller P Schaub S De Geest S Prevalence, consequences, and determinants of nonadherence in adult renal transplant patients: A literature review Transpl Int 2005 18 10 1121 1133 10.1111/j.1432-2277.2005.00176.x 16162098

[B3] Dew MA DiMartini AF De Vito Dabbs A Myaskovsky L Steel J Unruh M Switzer GE Zomak R Kormos RL Greenhouse JB Rates and risk factors for nonadherence to the medical regimen after adult solid organ transplantation Transplantation 2007 83 7 858 873 10.1097/01.tp.0000258599.65257.a6 17460556

[B4] De Geest S Denhaerynck K Dobbels F Grinyó JM Clinical and economic consequences of non-adherence to immunosuppressive drugs in adult solid organ transplantation. Compliance in solid organ transplantation International Transplantation Updates 2011 Barcelona, Spain: Permanyer Publications 63 81 [Invited Editor: Dr. Federico Oppenheimer]

[B5] Sabaté E Adherence to long-term therapies: Evidence for action 2003 Geneva: World Health Organization

[B6] Fine RN Becker Y De Geest S Eisen H Ettenger R Evans R Rudow DL McKay D Neu A Nevins T Reyes J Wray J Dobbels F Nonadherence consensus conference summary report Am J Transplant 2009 9 1 35 41 19133930 10.1111/j.1600-6143.2008.02495.x

[B7] Transplant Procurement Management - TPM 2011 [http://www.tpm.org]

[B8] Registro Brasileiro de Transplante[http://www.abto.com.br]

[B9] Tedesco HS Jr Felipe CR Abbud-Filho M Garcia V Medina-Pestana JO The Emerging Role of Brazil in Clinical Trial Conduct for Transplantation Am J Transplant 2011 11 7 1368 1375 10.1111/j.1600-6143.2011.03564.x 21668630

[B10] Denhaerynck K Burkhalter F Schäfer-Keller P Steiger J Bock A De Geest S Clinical consequences of non adherence to immunosuppressive medication in kidney transplant patients Transpl Int 2009 22 4 441 446 10.1111/j.1432-2277.2008.00820.x 19144090

[B11] Butler JA Roderick P Mullee M Mason JC Peveler RC Frequency and impact of nonadherence to Immunosuppressants after renal transplantation: a systematic review Transplantation 2004 77 5 769 789 10.1097/01.TP.0000110408.83054.88 15021846

[B12] De Geest S Burkhalter H De Bleser L Horne R Mason JC Non-adherence to immunosuppressive drugs in transplantation: What can clinicians do? Journal of Renal Nursing 2010 2 58 63

[B13] Osterberg LB Drug therapy: adherence to medication N Engl Med 2005 353 5 487 497 10.1056/NEJMra050100 16079372

[B14] Dobbels F Lut B De Geest S Drent G Lennerling A Whittaker C Kugler C Transplant360 Task Force The psychometric properties and practicability of self-report instruments to identify medication non-adherence in adult transplant patients to date: a systematic review Transplantation 2010 90 2 205 219 10.1097/TP.0b013e3181e346cd 20531073

[B15] Schmiid-Mohler G Thut MP Wüthrich RP Denhaerynck K De Geest S Non-adherence to immunosuppressive medication in renal transplant recipients within the scope of the integrative model of behavioral prediction: a cross-sectional study Clin Transplant 2009 10 213 222 10.1111/j.1399-0012.2009.01056.x 19674014

[B16] Transplant360 Task Force[http://www.transplant360.com]

[B17] Guillemin F Cross-cultural adaptation and validation of health status measures Scand J Rheumatol 1995 24 2 61 63 10.3109/03009749509099285 7747144

[B18] Guillemin F Bombardier C Beaton D Cross-cultural adaptation of health- related quality of life measures: literature review and proposed guidelines J Clin Epidemiol 1993 46 12 1417 1432 10.1016/0895-4356(93)90142-N 8263569

[B19] Terwee CB Bot SD de Boer MR van der Windt DA Knol DL Dekker J Bouter LM de Vet HC Quality criteria were proposed for measurement properties of health status questionnaires J Clin Epidemiol 2007 60 1 34 42 10.1016/j.jclinepi.2006.03.012 17161752

[B20] Jones PS Jerry WL Linda RP Zhang XE Jaceldo KB An adaptation of Brislin’s translation model for cross-cultural research Nurs Res 2001 50 5 300 304 10.1097/00006199-200109000-00008 11570715

[B21] Walsh JC Mandalia S Gazzard BG Responses to a 1 month self-report on adherence to antiretroviral therapy are consistent with electronic data and virological treatment outcome AIDS 2002 16 2 269 277 10.1097/00002030-200201250-00017 11807312

[B22] Kimberlin CL Winterstein AG Validity and reliability of measurement instruments used in research Am J Health Syst Pharm 2008 65 23 2276 2284 10.2146/ajhp070364 19020196

[B23] DeVellis RF Classical test theory Med Care 2006 44 Suppl 3 50 59 10.1097/01.mlr.0000245426.10853.30 17060836

[B24] Cronbach LJ Coefficient alpha and the internal structure of tests Psychometrika 1951 16 297 334 10.1007/BF02310555

[B25] Kelly WD, Ratliff JTA, Nedadic C Basic Statistics for Laboratories 1992 New York: Van Nostrand Reinhold

[B26] Delgado AB Lima ML Contributo para a validação concorrente de uma medida de adesão aos tratamentos Psicologia: Saúde e Doenças 2001 2 2 81 10022269375

[B27] Floyd JF Widaman KF Factor analysis in the development and refinement of clinical assessment instruments Psychol Assess 1995 7 286 289

[B28] Medina-Pestana JO Organization of a high-volume kidney transplant program - the “assembly line” approach Transplantation 2006 81 11 1510 1520 10.1097/01.tp.0000214934.48677.e2 16770238

[B29] Sousa SR Galante NZ Barbosa DA Pestana JOM Incidência e fatores de risco para complicações infecciosas no primeiro ano após o transplante renal Braz J Nephrol 2010 32 1 77 84

[B30] Yusuf H Gherunpong S Sheiham A Tsakos G Validation of an English version of the Child-OIDP index, an oral health-related quality of life measure for children Health Qual Life Outcomes 2006 4 38 10.1186/1477-7525-4-38 16813660 PMC1533817

[B31] Bengtsson M Ohlsson B Ulander K Development and psychometric testing of the Visual Analogue Scale for Irritable Bowel Syndrome (VAS-IBS) BMC Gastroenterol 2007 7 16 10.1186/1471-230X-7-16 17475020 PMC1868742

[B32] Varoli FK Pedrazzi V Adapted version of the McGill pain questionnaire to Brazilian Portuguese Braz Dent J 2006 17 4 328 335 17262148 10.1590/s0103-64402006000400012

[B33] Chisholm MA Lance CE Williamson GM Mulloy LL Development and validation of an immunosuppressant therapy adherence instrument (ITAS) Patient Educ Couns 2005 59 1 13 20 10.1016/j.pec.2004.09.003 16198214

[B34] Morisky D Green L Levine D Concurrent and predictive validity of a self reported measure of medication adherence Medical Care 1986 249 1 67 74 3945130 10.1097/00005650-198601000-00007

[B35] Schäfer-Keller P Steiger J Bock A Denhaerynck K De Geest S Diagnostic accuracy of measurement methods to assess non-adherence to immunosuppressive drugs in kidney transplant recipients Am J Transplant 2008 8 3 616 626 10.1111/j.1600-6143.2007.02127.x 18294158

[B36] Liu H Golin CE Miller LG Hays RD Beck CK Sanandaji S Christian J Maldonado T Duran D Kaplan AH Wenger NS A comparison study of multiple measures of adherence to HIV protease inhibitors Ann Intern Med 2001 134 10 968 977 10.7326/0003-4819-134-10-200105150-00011 11352698

[B37] Urquhart J Vrijens B New findings about patient adherence to prescribed drug dosing regimens: An introduction to pharmionics Eur J Hosp Pharm 2005 11 103 106

[B38] Uysal-Bozkir O Parlevliet JL de Rooij SE Insufficient cross-cultural adaptations and psychometric properties for many translated health assessment scales: A systematic review J Clin Epidemiol 2013 U U Feb 15. Epub ahead of print 10.1016/j.jclinepi.2012.12.004 23419610

[B39] Morrissey EP Flynn LM Lin S Medication noncompliance and its implications in transplant recipients Drugs 2007 67 10 1463 1481 10.2165/00003495-200767100-00007 17600393

[B40] Pinsky BW Takemoto SK Lentine KL Burroughs TE Schnitzler MA Salvalaggio PR Transplant outcomes and economic costs associated with patient noncompliance to immunosuppression Am J Transplant 2009 9 11 2597 2606 10.1111/j.1600-6143.2009.02798.x 19843035

